# Differential Expression of Type III Effector BteA Protein Due to IS*481* Insertion in *Bordetella pertussis*

**DOI:** 10.1371/journal.pone.0017797

**Published:** 2011-03-10

**Authors:** Hyun-Ja Han, Asaomi Kuwae, Akio Abe, Yoshichika Arakawa, Kazunari Kamachi

**Affiliations:** 1 Department of Bacteriology II, National Institute of Infectious Diseases, Tokyo, Japan; 2 Laboratory of Bacterial Infection, Kitasato Institute for Life Sciences, Kitasato University, Tokyo, Japan; Institut de Pharmacologie et de Biologie Structurale, France

## Abstract

**Background:**

*Bordetella pertussis* is the primary etiologic agent of the disease pertussis. Universal immunization programs have contributed to a significant reduction in morbidity and mortality of pertussis; however, incidence of the disease, especially in adolescents and adults, has increased in several countries despite high vaccination coverage. During the last three decades, strains of *Bordetella pertussis* in circulation have shifted from the vaccine-type to the nonvaccine-type in many countries. A comparative proteomic analysis of the strains was performed to identify protein(s) involved in the type shift.

**Methodology/Principal Finding:**

Proteomic analysis identified one differentially expressed protein in the B. pertussis strains: the type III cytotoxic effector protein BteA, which is responsible for host cell death in Bordetella bronchiseptica infections. Immunoblot analysis confirmed the prominent expression of BteA protein in the nonvaccine-type strains but not in the vaccine-type strains. Sequence analysis of the vaccine-type strains revealed an IS481 insertion in the 5′ untranslated region of bteA, −136 bp upstream of the bteA start codon. A high level of bteA transcripts from the IS481 promoter was detected in the vaccine-type strains, indicating that the transcript might be an untranslatable form. Furthermore, BteA mutant studies demonstrated that BteA expression in the vaccine-type strains is down-regulated by the IS481 insertion.

**Conclusion/Significance:**

The cytotoxic effector BteA protein is expressed at higher levels in B. pertussis nonvaccine-type strains than in vaccine-type strains. This type-dependent expression is due to an insertion of IS481 in B. pertussis clinical strains, suggesting that augmented expression of BteA protein might play a key role in the type shift of B. pertussis.

## Introduction

*Bordetella pertussis* is a human-specific pathogen that is the etiologic agent of whooping cough, an acute respiratory disease that is often particularly severe in infants [1]. Universal immunization programs have contributed to a significant reduction in morbidity and mortality of pertussis, especially in infants and children; however, the incidence of pertussis has increased in several countries despite high vaccination coverage [2]–[5]. Since the 1980s, a considerable genetic transition has been observed between *B. pertussis* vaccine strains and circulating clinical strains in many countries [6]–[11]. Genetic variations have been found in the loci encoding the major *B. pertussis* virulence factors: pertussis toxin S1 subunit (*ptxA*), pertactin (*prn*) and fimbriae 3 (*fim3*). Among circulating *B. pertussis* strains, vaccine-type alleles (*ptxA2*, *prn1* and *fim3A*) have been replaced mainly with nonvaccine-type alleles (*ptxA1*, *prn2* and *fim3B*). It has been speculated that adaptation of the bacterial population to vaccine-induced immunity has produced this genetic shift, and is one possible explanation for the resurgence of pertussis [12]–[15]. However, there have been few reports of the exact mechanism underlying this phenomenon.

*B. pertussis* expresses various virulence factors, including adhesins and toxins, which function to establish and maintain host infection. Several virulence factors such as filamentous haemagglutinin (FHA) and pertussis toxin (PT) are expressed under the control of the BvgAS two-component regulatory system [1], [16], [17]. The BvgAS system also positively regulates virulence factor secretion via the type III secretion system (T3SS) [18], [19]. T3SS is highly conserved among a number of Gram-negative bacteria and functions as an injector of virulence molecules (i.e., effectors) into the host cell through a needle-like injection apparatus [20], [21]. In *B. pertussis*, T3SS plays a role in subverting the protective innate and adaptive immunity of the host. Three T3SS-secreted proteins, BopN, BopD and Bsp22, have been identified so far [22]. In the animal pathogen *Bordetella bronchiseptica*, BopN is involved in the up-regulation of cytokine IL-10 [23], while Bsp22 polymerizes to form a flexible filamentous structure at the tip of the needle structure and associates with the pore component BopD [24]. The Bsp22 translocon is expressed in a significant proportion of *B. pertussis* clinical isolates but not in Tohama and Wellcome 28, the common laboratory-adapted vaccine strains [22].

Genomic differences between *B. pertussis* clinical strains and the vaccine strain Tohama have been investigated. The comparative genomics profiling revealed that the genome of *B. pertussis* Tohama differs from clinical isolates in four regions (RD11 to RD14) [25]. In contrast, progressive gene loss mediated by homologous recombination between IS*481* insertion sequence elements has been observed among recently circulating strains of *B. pertussis* isolates [26], [27]. IS*481* is present in multiple copies on the *B. pertussis* chromosome, and it plays a critical role in *B. pertussis* evolution through genomic rearrangement.

Proteomic analysis has been widely applied to comparisons of protein expression among different strains, and information accumulated from genomic studies of *Bordetella* spp. facilitates comparative proteomic approaches to the investigation of *B. pertussis* clinical strains [6], [28]. In the present study, a proteomic approach was employed to identify the protein(s) involved in the genetic shift from vaccine-type to nonvaccine-type in *B. pertussis* strains. The protein profile analyses identified one differentially expressed protein, the T3SS effector BteA (alias BopC) [29], [30], between the strain types. BteA is a 68 kDa cytotoxic effector that has been identified in *B. bronchiseptica* but not in the *B. pertussis* human pathogen. Here we studied the differential expression of BteA protein in *B. pertussis* clinical strains and identified a specific IS*481* insertion in the 5′ untranslated region (5′-UTR) of *bteA* in vaccine-type strains.

## Results

### Identification of BteA in *B. pertussis* nonvaccine-type strain

**Figure 1:**
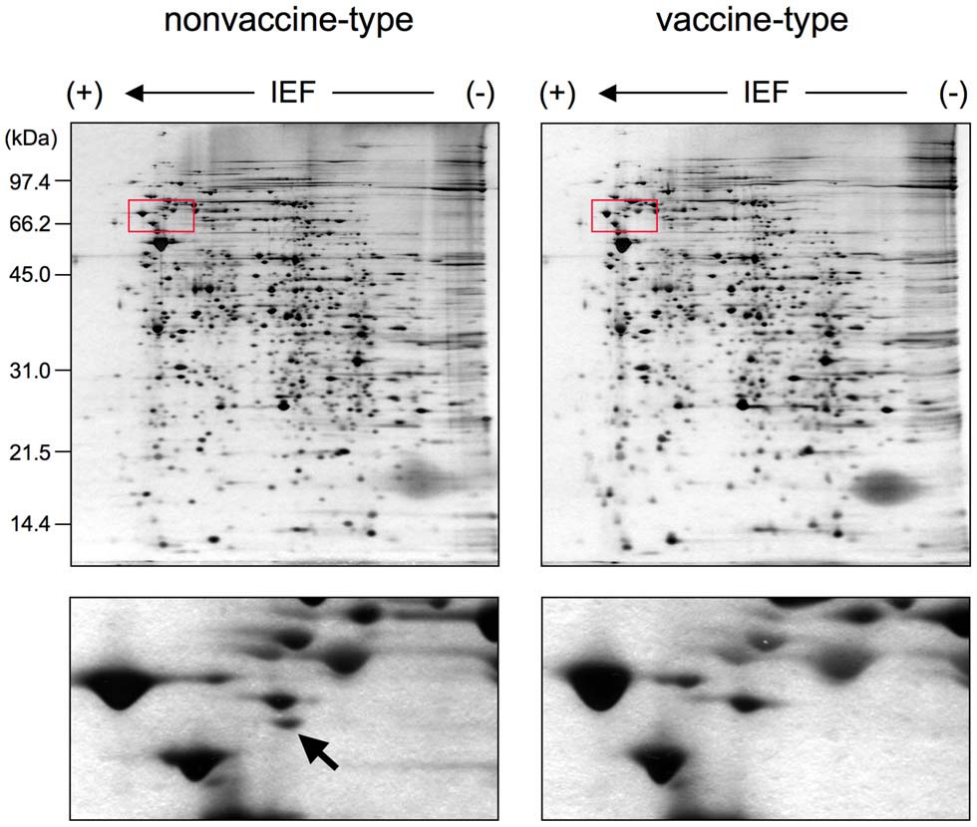
Comparative proteomic analysis of *B. pertussis* nonvaccine-type and vaccine-type strains. Total protein (10 µg) from the nonvaccine-type and vaccine-type clinical strains was separated by 2-D gel electrophoresis and silver stained. The left upper panel shows the protein profile of the nonvaccine-type BP235. The right upper panel shows the protein profile of the vaccine-type BP233. The red-boxed areas are enlarged (lower panels). The arrow in left lower panel indicates the spot that was identified as type III effector BteA by LC-MS/MS analysis.

A comparative proteomic analysis of two clinical strains was performed to investigate the shift of *B. pertussis* strains from vaccine-type to nonvaccine-type. Figure 1 shows 2-dimensional electrophoretic (2-DE) maps of total protein expressed in the nonvaccine-type clinical strain BP235 and the vaccine-type BP233. Among >600 protein spots detected on the 2-DE gel, one was notably absent in the 2-DE map of BP233. The protein spot was observed in other nonvaccine-type strains (BP157, BP159, BP162 and BP228), but not in other vaccine-type strains (BP155, BP156, BP232 and BP243). The protein represented by the spot was identified by LC-MS/MS analysis using tryptic digests. The MS/MS of the protein digests provided four peptide sequences (RPDEFAAR, FDALR, ITALNLR and TQTQLLALQR) that matched the *B. pertussis* hypothetical protein BP0500 (NCBI accession: NP_879352). Hypothetical protein BP0500 was identified as the T3SS effector BteA, since the sequence is highly conserved with 98% amino acid identity to the BteA (BopC) of *B. bronchiseptica* [29], [30].

### High expression of BteA protein in nonvaccine-type strains

**Figure 2:**
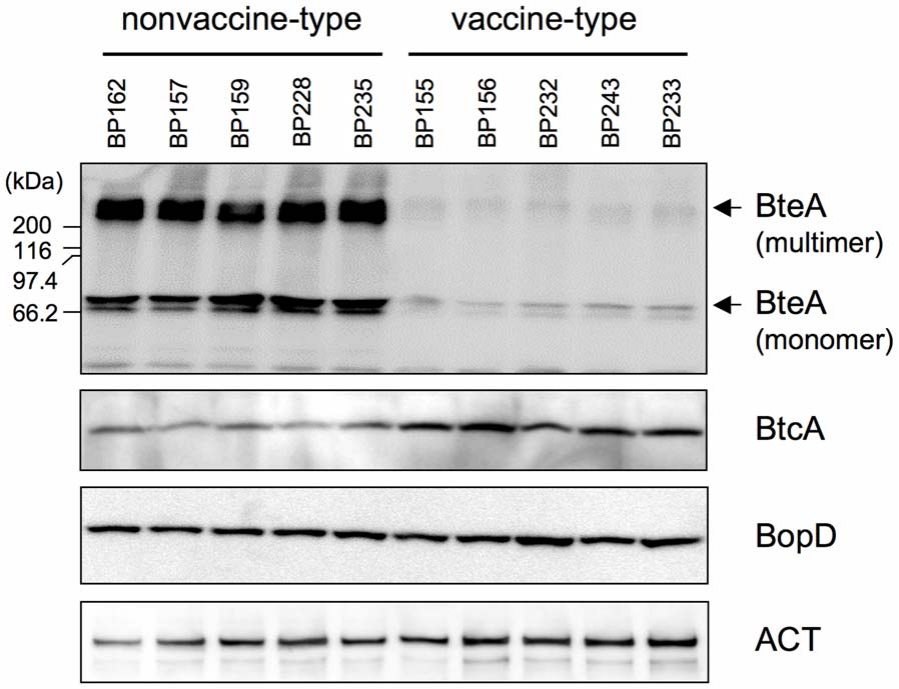
Expression of BteA, BtcA, BopD and ACT proteins in *B. pertussis* nonvaccine-type and vaccine-type strains. The nonvaccine-type clinical strains (BP157, BP159, BP162, BP228 and BP235) and vaccine-type clinical strains (BP155, BP156, BP232, BP233 and BP243) were cultured in modified SS medium for 18 h. Total protein extracted from bacterial cells was subjected to immunoblot analysis with anti-BteA, anti-BtcA, anti-BopD or anti-ACT antiserum. For BteA detection, 10 µg of total protein was loaded in each lane.

Immunoblots of *B. pertussis* clinical strains using anti-BteA antiserum detected high levels of a protein of ∼68 kDa in all nonvaccine-type clinical strains (BP157, BP159, BP162, BP228 and BP235), whereas BteA expression was greatly reduced in the vaccine-type clinical strains (BP155, BP156, BP232, BP233 and BP243). Additional products of >200 kDa were also detected in the nonvaccine-type clinical strains. These high molecular mass signals appear to be the protein bands that have been reported as a multimeric complex of BteA in *B. bronchiseptica* [29], [30] (see Figure S1). T3SS function in the nonvaccine-type strains was confirmed by using whole cell protein extracts for immunoblots of BtcA (the BteA chaperone) [29], [31] and BopD (the T3SS translocon) [32]. BtcA and BopD polypeptides were detected in both strain types, but the BtcA signals produced by the nonvaccine-type strains were apparently lower than those of the vaccine-type strains (Figure 2). The reason for the different expression is not clear. In contrast, adenylate cyclase toxin (ACT), another *Bordetella* spp. virulence factor, was detected at similar levels in both strain types.

**Figure 3:**
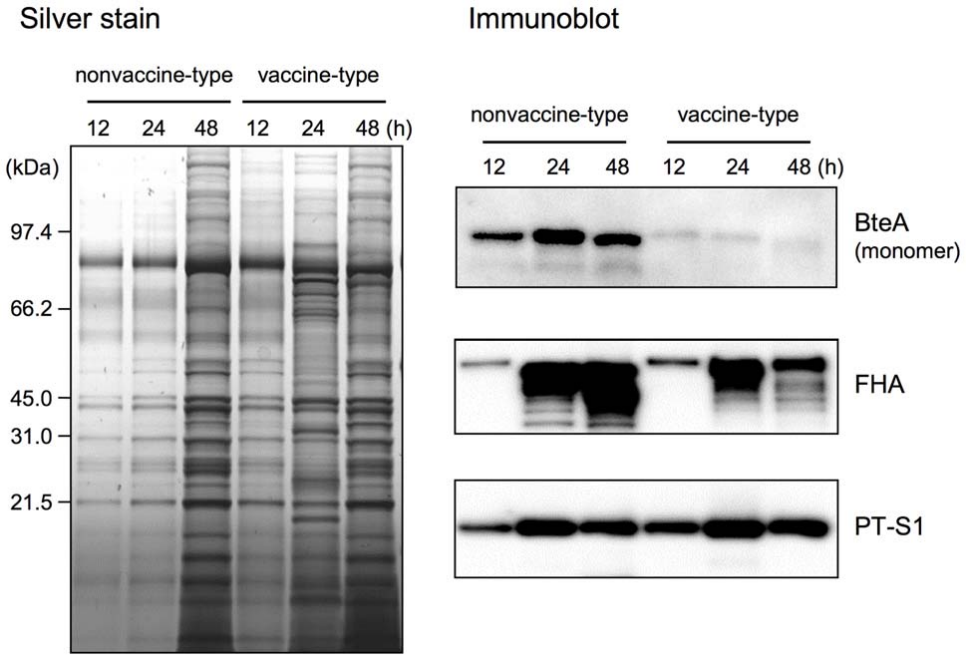
BteA secretion from *B. pertussis* nonvaccine-type and vaccine type strains. Strains BP235 (nonvaccine-type) and BP233 (vaccine-type) were cultured in modified SS medium, and the culture supernatants (CS) were collected at 12, 24 and 48 h. Protein samples prepared by precipitation with 10% trichloroacetic acid were separated by 12.5% SDS-PAGE followed by silver staining (left panel). BteA, FHA and PT secretions were analyzed by immunoblots using anti-BteA, anti-FHA or anti-PT antiserum (right panels). For BteA detection, the equivalent of 200 µl of CS was loaded in each lane.

In order to confirm BteA secretion by *B. pertussis* strains, BteA polypeptide in the culture supernatants (CS) was subjected to immunoblot analysis. BteA was detected in secreted proteins from the nonvaccine-type clinical strain BP159 at 12, 24 and 48 h, whereas the signal was very low in the vaccine-type clinical strain BP155 over the 48-h time period (Figure 3). Conversely, signals corresponding to PT-S1 subunit and FHA polypeptides were detected in the supernatants of both cultures throughout the sampling period, although silver staining revealed small differences in their protein profiles after 24 h in culture.

### Transcription of *bteA*

**Figure 4:**
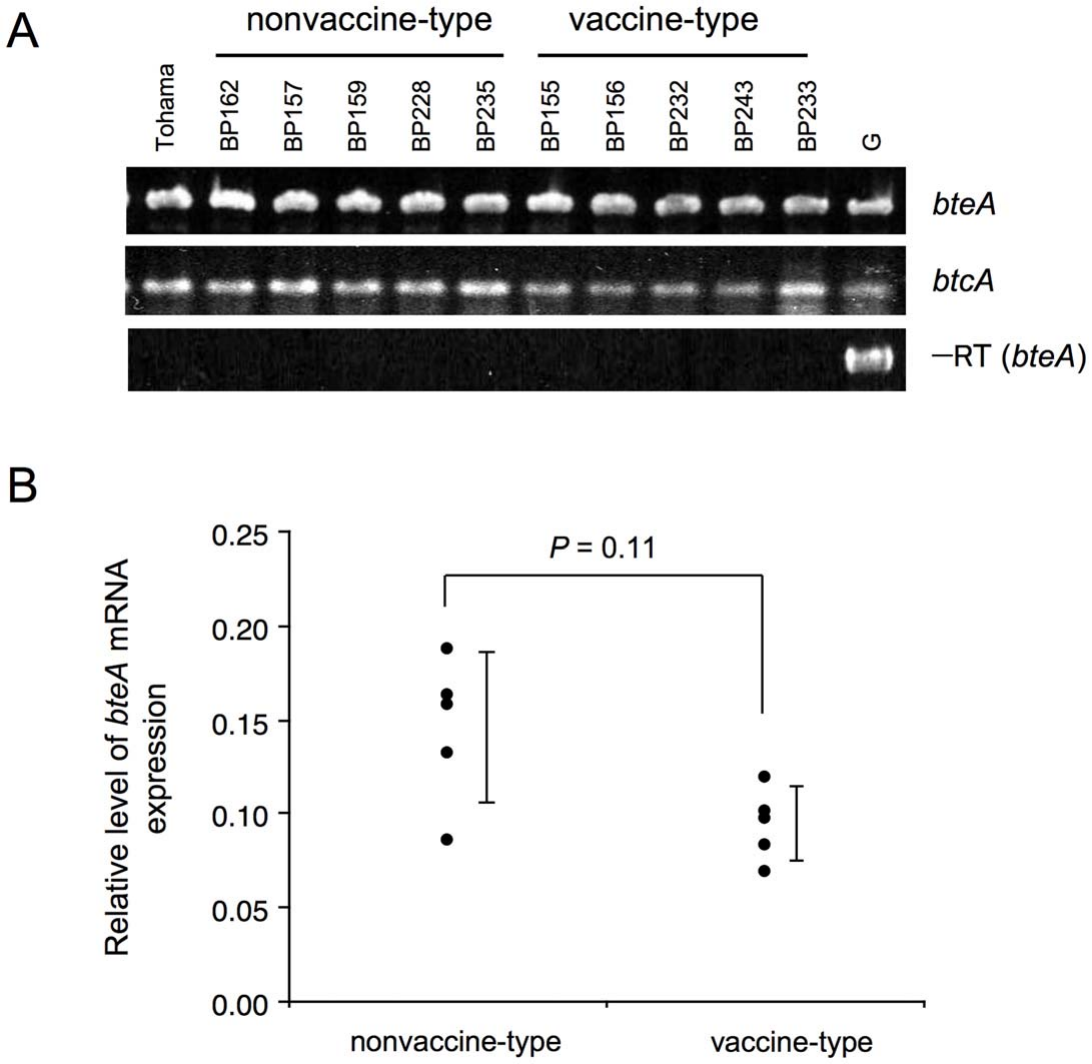
RT-PCR analysis of *bteA* transcript in *B. pertussis* nonvaccine-type and vaccine-type strains. (A) RT-PCR with primers specific for *bteA* and *btcA*. cDNA made from total RNA of nonvaccine-type (BP157, BP159, BP162, BP228 and BP235) and vaccine-type (BP155, BP156, BP232, BP233 and BP243) clinical strains was used as templates for PCR. Genomic DNA (G) from *B. pertussis* strain Tohama was used as a positive control. A mock reaction for *bteA* (−RT) consisted of reactions lacking reverse transcriptase. (B) Quantitative RT-PCR analysis of *bteA* transcript levels in the nonvaccine-type and vaccine-type clinical strains listed in (A). The *recA* transcript was used as a reference. Each point represents one strain and vertical bars indicate standard deviations.

*bteA* gene expression in *B. pertussis* strains was investigated with conventional RT-PCR and quantitative RT-PCR. *bteA* was transcribed in both the nonvaccine-type (BP157, BP159, BP162, BP228 and BP235) and vaccine-type (BP155, BP156, BP232, BP233 and BP243) clinical strains (Figure 4A). Similarly, *btcA* transcripts were detected in both strain groups. RT-PCR experiments lacking reverse transcriptase showed no specific product for *bteA* amplification, confirming negligible genomic DNA contamination in the RNA preparations. Quantitative RT-PCR (qRT-PCR) showed an average *bteA* transcript level of 0.146 (±1SD range, 0.107 to 0.184) in nonvaccine-type strains and 0.095 (±1SD range, 0.076 to 0.113) in vaccine-type clinical strains, a difference that was not statistically significant (*P* = 0.11) (Figure 4B).

### IS*481* insertion in the *bteA* 5′-UTR in vaccine-type strains

**Figure 5:**
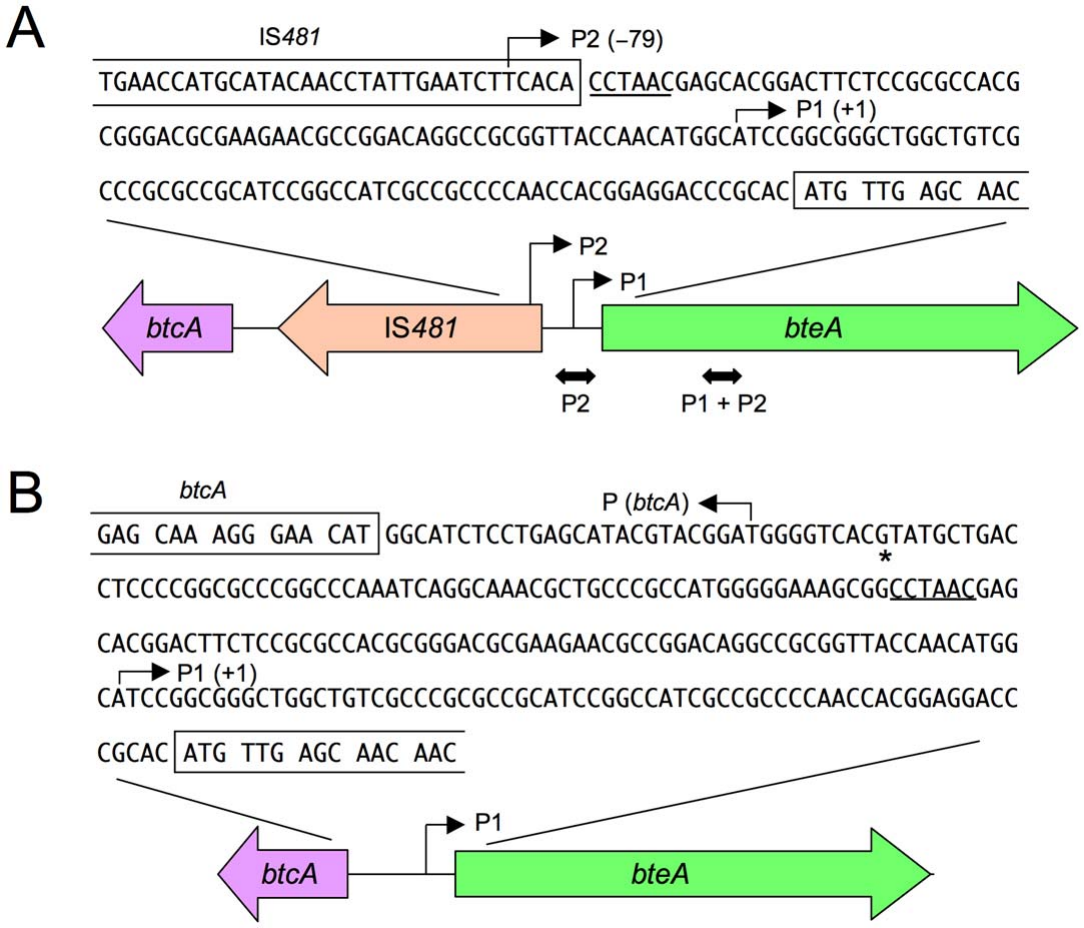
Physical maps of the *btcA−bteA* region of *B. pertussis* vaccine-type and nonvaccine-type strains. (A) The vaccine-type clinical strain BP155. The location of IS*481a* is represented by a gray arrow on the physical map. The recognition sequence of IS*481a* is underlined. The two mapped transcriptional start sites (P1 and P2) of *bteA* are shown by arrows. Region amplified by qRT-PCR to determine the IS*481a*-promoter (P2) and total (P1 + P2) transcripts are shown by two-headed arrows below the physical map. (B) The nonvaccine-type clinical strain BP159. The mapped transcriptional start sites of *bteA* (P1) and *btcA* [P (*btcA*)] are shown by arrows. The single nucleotide polymorphism (A→G) at −207 bp from the *bteA* translation start codon is indicated by an asterisk.

Sequencing of the *bteA* 5′-UTR of the five vaccine-type strains (BP155, BP156, BP232, BP233 and BP243), revealed a 1,043-bp insertion sequence (IS*481*) −136 bp upstream of the *bteA* start codon (Figure 5A). IS*481a*, which is newly identified in *B. pertussis*, showed 99% nucleotide sequence identity with IS*481* of *B. pertussis* Tohama. The CCTAAC sequence in the *bteA* 5′-UTR is an insertion site of IS*481a* and is duplicated by the insertion, although the 6-bp consensus recognition sequence of IS*481* has been reported as NCTAGN [33]. IS*481* insertions were not found in the nonvaccine-type clinical strains, which had nucleotide sequences that were 99% identical to that of *B. pertussis* Tohama. In the *bteA* 5′-UTR of the nonvaccine-type strains (BP157, BP159, BP162 BP228 and BP235), one single nucleotide polymorphism (A→G) was observed at 207 bp upstream of the *bteA* translation start site (Figure 5B).

The *bteA* 5′-UTR was PCR-amplified from chromosomal DNA of other *B. pertussis* strains to confirm insertion of IS*481*. Among 61 vaccine-type clinical strains, 60 (98%) produced amplicons of ∼3.1 kb, a size indicative of an IS*481* insertion in the *bteA* 5′-UTR. One strain (BP121) had a product of ∼2.1 kb, corresponding to the predicted size of the native 5′-UTR (data not shown). Of the 23 nonvaccine-type strains examined, all generated ∼2.1 kb amplicons, confirming the absence of the IS*481* insertion.

### Determination of the *bteA* transcription start site

5′-RACE mapping was used to identify the *bteA* transcription start site in vaccine-type strain BP155. Nucleotide sequences of the 5′-RACE PCR products revealed two transcription start sites, P1 and P2, located −68 and −147 bp from the *bteA* translation start codon (Figure 5A). The P1 start site (+1) was located within the *bteA* 5′-UTR, whereas the P2 start site (−79) was located within IS*481a*. Only the P1 start site was also found in the nonvaccine-type strain BP159 (Figure 5B). IS*481* contains an outward-facing promoter at one end that is responsible for transcription of the flanking catalase gene (*katA*) in *B. pertussis* [34]. However, the P2 start site is different from the *katA* transcription start site. The transcription start site of *btcA*, also determined by 5′-RACE, was mapped to a T residue 31 bp upstream of the *btcA* translation start codon in both the vaccine-type and nonvaccine-type strains (Figure 5B).

Primer extension analysis was also performed in an attempt to resolve the *bteA* transcription start sites. However, the start sites could not be ascertained, probably due to low amounts of *bteA* transcript in *B. pertussis*.

### IS*481a*-promoter transcript is the major *bteA* transcript in the vaccine-type strain

Expression of the IS*481a*-promoter transcript (P2 transcript) in *B. pertussis* vaccine-type strain BP155 was analyzed by qRT-PCR with TaqMan probes (Figure 5A). The P2 transcript and total *bteA* (P1 + P2) transcripts were determined individually and the ratio of P2 transcript to total *bteA* transcript was calculated. Based on four independent experiments, the ratio (P2 transcript/P1 + P2 transcripts) was estimated to be 0.88 (±1SD range, 0.70 to 1.09), indicating that the P2 transcript is the major *bteA* transcript in the vaccine-type strain (data not shown).

### BteA expression in *B. pertussis* BteA mutants

**Figure 6:**
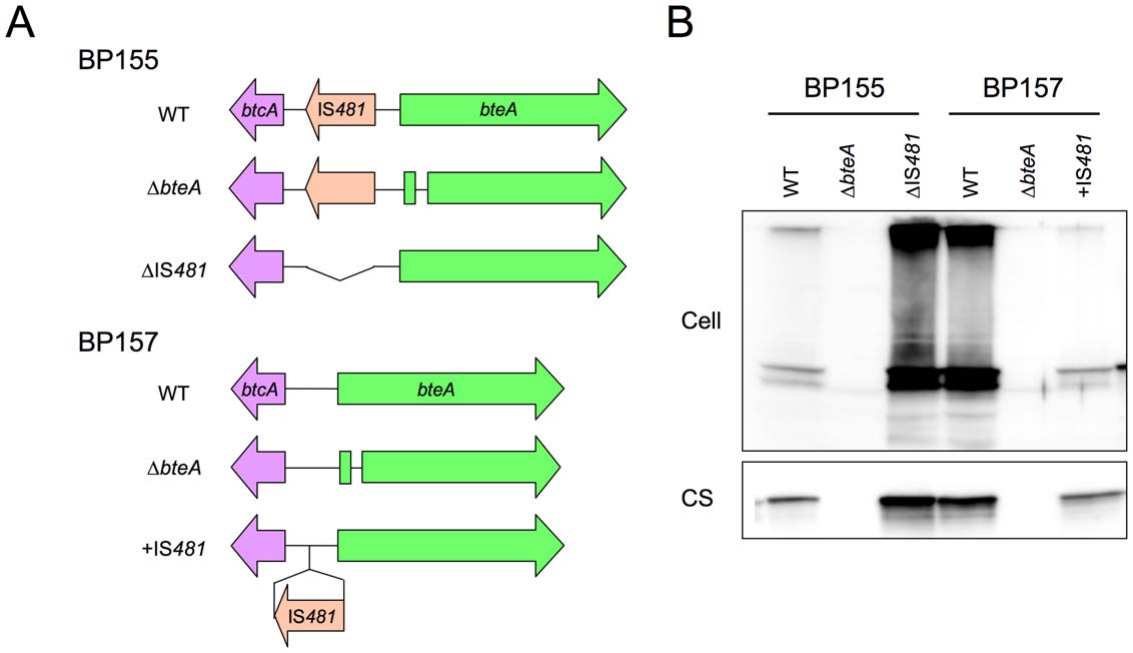
Construction and characterization of *B. pertussis* BteA mutants. (A) Physical map of the *btcA−bteA* region of BteA mutants derived from *B. pertussis* BP155 (vaccine-type) and BP157 (nonvaccine-type). WT, wild-type; Δ*bteA*, a 178-bp deletion around the 5′ region of *bteA*; ΔIS*481*, IS*481a* deletion from the *bteA* 5′-UTR; +IS*481*, IS*481a* insertion in the *bteA* 5′-UTR. (B) Expression of BteA protein in the BteA mutants. The mutants were cultured in modified SS medium for 24 h. Total protein from the bacterial cells (Cell) and culture supernatants (CS) was analyzed with immunoblot using anti-BteA antiserum.

To clarify the effect of the IS*481* insertion on BteA expression, four BteA mutants (Δ*bteA*-BP155, ΔIS*481*-BP155, Δ*bteA*-BP157 and +IS*481*-BP157) were constructed from *B. pertussis* BP155 (vaccine-type) and BP157 (nonvaccine-type) by homologous recombination (Figure 6A). The Δ*bteA*-BP155 and Δ*bteA*-BP157 mutants had a 178-bp deletion in the 5′ region of *bteA*. In the ΔIS*481*-BP155 mutant, a 2.2-kb insertion containing an intact *bteA* 5′-UTR (derived from *B. pertussis* Tohama) replaced the native *bteA* 5′-UTR + IS*481a* gene. In contrast, +IS*481*-BP157 mutant had a 3.2-kb insertion containing a *bteA* 5′-UTR + IS*481a* (derived from *B. pertussis* BP155) instead of its own *bteA* 5′-UTR. Consequently, ΔIS*481*-BP155 had an IS*481a* deletion from the *bteA* 5′-UTR, whereas the +IS*481*-BP157 mutant had an IS*481a* insertion in the *bteA* 5′-UTR. The *btcA−bteA* region of the mutants was verified by DNA sequence analysis.

BteA expression in the bacterial cells and CS after 24 h in culture was analyzed by immunoblot with anti-BteA antiserum (Figure 6B). In ΔIS*481*-BP155 bacterial cells and CS, BteA polypeptide(s) corresponding to ∼68 kDa and >200 kDa were detected at the same level as was observed in the BP157 wild-type strain. In contrast, the signals of BteA polypeptide(s) from +IS*481*-BP157 mutant were very low in both bacterial cells and CS. Similarly, BteA polypeptide(s) were not detected in either Δ*bteA*-BP155 or Δ*bteA*-BP157. These results clearly showed that BteA protein expression is down-regulated by the IS*481* insertion in *B. pertussis*, and that the anti-BteA antiserum is highly specific to BteA.

## Discussion

The BteA effector (alias BopC) is required for the induction of necrotic cell death during *B. bronchiseptica* infections, and is thought to play a pivotal role in T3SS-mediated cell death [29], [30], [35]. BteA is also involved in dephosphorylation of tyrosine-phosphorylated proteins (PY) of host cells [30], and its 130-amino acid N-terminal sequence is associated with target lipid rafts [31]. BteA is the only cytotoxic effector that has been identified in *Bordetella* spp. In *B. pertussis*, low-passage clinical strains have an ability to express a functionally active T3SS; however, BteA protein had not been detected in the clinical and common laboratory-adapted strains by MALDI-TOF mass spectrometry [22]. Here we demonstrate that BteA protein is highly expressed in *B. pertussis* nonvaccine-type strains but not in the vaccine-type strains, and that BteA protein expression is down-regulated by IS*481a* insertion in the vaccine-type strains. We provide the first evidence that BteA protein expression is type-dependent due to the IS*481a* insertion in *B. pertussis* clinical strains.

In Japan, *B. pertussis* circulating strains began to change from vaccine-type to nonvaccine-type in the mid-1990s [8], and the reported incidence of adult cases of pertussis has dramatically increased since 2002 [36]. The genetic divergence in *B. pertussis* circulating strains has also been observed in many other countries. A possible explanation for the genetic divergence is that the type shift is a result of vaccine-driven evolution [12]–[15]. More recently, Mooi et al. [37] reported that expansion of *B. pertussis* strains with increased PT production has contributed to the resurgence of pertussis in the Netherlands. Here we showed prominent expression of the T3SS effector protein BteA in the nonvaccine-type strains, and that PT and ACT (important virulence factors of *B. pertussis*) are expressed at the same level in both the nonvaccine and vaccine-type strains. Besides vaccine-driven evolution, our findings could provide another possible explanation for the type shift from vaccine-type to nonvaccine-type, i.e., the augmented expression of BteA protein in *B. pertussis* nonvaccine-type strains may be involved in the type shift.

*B. bronchiseptica* BteA has *in vitro* cytotoxic activity against cultured mammalian cells [18], [22], [29], [30]. In this study, we determined the cytotoxicity of *B. pertussis* BteA mutants by measuring the release of lactate dehydrogenase (LDH) from L2 rat lung epithelial cells, J774 mouse macrophage-like cells, or HeLa cells. However, even BteA-expressing strains (ΔIS*481*-BP155 and wild-type BP157) showed low cytotoxicity (<10%), and consequently no statistically significant differences in cytotoxicity were observed among the wild-type and mutant strains. *B. pertussis* is known to have a lower *in vitro* cytotoxicity than *B. bronchiseptica* [18], [22], which is consistent with the extremely low secretion of BteA in *B. pertussis* as compared to *B. bronchiseptica* (Figure S1). Therefore, a more sensitive and quantitative assay is required to determine the BteA-dependent cytotoxicity of *B. pertussis*.

IS*481* belongs to the recently defined IS*481* family [38], and 238 copies of IS*481* are present in the *B. pertussis* Tohama genome [39]. In *B. pertussis* clinical strains, IS*481* is also present in multiple copies on the chromosome and it plays a critical role in *B. pertussis* evolution [26], [40]. Many IS elements have been shown to activate the expression of neighboring genes. IS*481* contains an outward-facing promoter that is located in close proximity to the left terminal inverted repeat, and this promoter is responsible for the transcription of *katA* in certain *B. pertussis* strains [34]. Here we identified an IS*481a* insertion in the *bteA* 5′-UTR in *B. pertussis* vaccine-type clinical strains and detected a high level of *bteA* transcripts from the IS*481a* promoter (P2) compared with its own promoter (P1). However, the vaccine-type strains showed a low level of BteA protein expression, suggesting that insertion of IS*481a* represses P1 promoter activity, and that P2 transcript has a low translational efficiency from the additional nucleotide sequence (79 nucleotides) at its 5′ end. Use of a cell-free coupled transcription-translation system revealed that the additional nucleotide sequence is involved in down-regulation of transcription and/or translation (Figure S2). The 5′-UTR of bacterial mRNAs can bear regulatory elements that are involved in down- or up-regulation of translation [41]. The regulatory mechanisms in this region are controlled by RNA-binding proteins, small noncoding RNAs and structural rearrangements with the 5′-UTR. In addition, a 5′ stem-loop structure that sequesters the ribosomal binding site has been shown to be involved in translational regulation. Bioinformatic analysis uncovered a predicted stem-loop structure in the *bteA* 5′-UTR of P2 transcript (Figure S2).

In this study, the 5′-UTRs of five *B. pertussis* vaccine-type clinical strains were sequenced; all had an insertion of an IS*481a* in the *bteA* 5′-UTR, both transcribed in the same direction. In one of the vaccine-type strains, BP155, the major *bteA* mRNA was transcribed from P2 in the IS*481a*-promoter. These observations raise the possibilities that (i) the P2 transcript is translated into BteA under certain environmental conditions, and (ii) the P2 transcript is translated into another novel protein by translational frameshifting. BteA is known to be regulated by the BvgAS system and an extracytoplasmic function (ECF) sigma factor BtrS in *B. bronchiseptica* [18], [29]. In *B. pertussis*, it has been suggested that expression of the T3SS translocon Bsp22 is blocked by post-transcriptional regulation [18]. However, the molecular details of the regulatory mechanism are still unclear. Further studies are needed to determine the down-regulation of BteA protein in *B. pertussis* vaccine-type clinical strains.

In conclusion, *B. pertussis* vaccine-type strains have been replaced with the nonvaccine-type strains in many countries, and the resurgence of pertussis has been observed in several nations. In Japanese *B. pertussis* clinical strains, the T3SS effector BteA is highly expressed in nonvaccine-type strains as compared with the vaccine-type strains. Our findings indicate that augmented expression of BteA protein in *B. pertussis* circulating strains could play a key role in the type shift. However, it is unclear whether BteA protein is implicated in the resurgence of pertussis. Further studies are needed to determine the expression of BteA protein in *B. pertussis* circulating strains on a global scale.

## Materials and Methods

### Bacterial strains and growth conditions

*B. pertussis* clinical strains were selected from the laboratory collection of the National Institute of Infectious Diseases, Tokyo, Japan. The selection criteria included the time and geographic location of isolation, and their *ptxA* and *prn* alleles. A total of 10 clinical strains from 2002 to 2004 in Japan were included. Of the 10 clinical strains, 5 harbored *ptxA1* and *prn2* alleles (BP157, BP159, BP162, BP228 and BP235; nonvaccine-type strains), while the others carried *ptxA2* and *prn1* (BP155, BP156, BP232, BP233 and BP243; vaccine-type strains). All strains were cultured on Bordet-Gengou agar (BG agar, Difco) supplemented with 1% glycerol and 15% defibrinated horse blood or in modified Stainer-Scholte (SS) medium [42].

### Two-dimensional gel electrophoresis (2D-PAGE)

2D-PAGE was performed based on the O'Farrell method [43] with minor modifications. *B. pertussis* clinical strains grown on BG agar plates were resuspended in casamino acid solution (1% casamino acid, 0.6% NaCl, pH 7.1). Bacterial cells were precipitated by centrifugation (12,000 × *g*, 10 min) and resuspended in SDS-lysis buffer (62.5 mM Tris-HCl, 1% SDS, 10% glycerol, 5% 2-mercaptoethanol, pH 6.8) by sonication. Total protein was extracted by boiling for 3 min, followed by centrifugation. A portion (10 µg, approximately 2 µl) of the protein solution was mixed with 20 µl of sample buffer [8.5 M urea, 2% Nonidet P-40, 2% Ampholine (pH 3.5 to 10)], and applied to an isoelectric focusing tube gel (2.0 mm inside diameter by 12.0 cm) containing 4% polyacrylamide, 8.5 M urea, 2% Nonidet P-40, and 2% Ampholine (pH 5 to 7 and pH 3.5 to 10 in a ratio of 1∶4). Proteins were focused at 10°C for 17 h (1 h at 200 V, 2 h at 400 V, and 14 h at 800 V) with 10 mM H_3_PO_4_ (anolyte) and 20 mM NaOH (catholyte). In the second dimension, the electrofocused tube gel was electrophoresed in 12% SDS-PAGE. The separated polypeptides were visualized by silver staining and analyzed with the PDQuest 2-D Analysis Software (Bio-Rad, Hercules, CA). The Lowry assay was used to measure protein concentrations in a trichloroacetic acid (TCA) pellet (resuspended in 1 N NaOH) using bovine serum albumin as a standard.

### Protein identification

2D-PAGE gels were stained with silver nitrate without glutaraldehyde fixation [44], and protein spots of interest were excised. Proteins were reduced with 10 mM DTT, alkylated with 55 mM iodoacetamide, and digested with sequencing grade-modified trypsin (Promega, Madison, WI). Digested peptides were separated on a C18 capillary column (0.2 by 50 mm, Michrom Bioresources, CA) equipped with a Chorus 220 solvent delivery system and an HTC PAL auto-sampler system (CTC Analytics AG, Zwingen, Switzerland). Separated peptides were analyzed by the Finnigan LCQ^TM^ Deca XP ion trap mass spectrometer (Thermo Fisher Scientific Inc., MA) with electrospray ionization (ESI) interface using the Nanosprayer FS (GL Sciences Inc., Japan). To identify peptides, data files were generated from the MS/MS scans by Bioworks 3.0 using the SEQUEST algorithm (threshold, 10^5^; minimum group scan 2, Xc >1.0, Thermo Fisher Scientific) and searched against the complete amino acid database derived from the *B. pertussis* Tohama genome database.

### Antibody production against recombinant BteA, BtcA and ACT

The BteA gene (NCBI accession: NP_879352) was amplified by PCR from *B. pertussis* Tohama DNA using BteA-F and BteA-R primers, and cloned into the XmnI/HindIII sites of pMal-c2X (New England Biolabs, Beverly, MA) to generate a maltose binding protein (MBP) fusion with BteA. Production of this fusion protein was induced in *E. coli* BL21 with 0.5 mM isopropyl-β-D-thiogalactopyranoside (IPTG) and subsequently purified using amylose resin (New England Biolabs) and Resource Q (Amersham Pharmacia Biotech, Uppsala, Sweden) columns. A two-step PCR was carried out to amplify recombinant BtcA (NCBI accession: NP_879351). The first PCR was performed using the BtcA-BteA-F3 and BtcA-BteA-R3 primers (Table S1), which amplified the region between positions 165122 and 167190 of the *B. pertussis* Tohama genome (GenBank accession: BX640412). In the second PCR, *btcA* was amplified from the first PCR product with the 5-BtcA and 3-BtcA primers (Table S1) and cloned into the NdeI/HindIII sites of pCold II DNA (TAKARA Bio Inc.). His-tagged BtcA was induced in *E. coli* BL21 with 0.5 mM IPTG at 15°C and purified using the HisTrap FF Crude Kit (GE Healthcare UK Ltd.). A recombinant catalytic domain of *B. pertussis* adenylate cyclase toxin (ACT) was a gift from Mineo Watanabe.

Antibodies against MBP-BteA, BtcA and ACT were generated in mice at Nippon Biotest Laboratories, Inc. (Tokyo, Japan). The MBP-BteA antiserum was pre-absorbed with MBP2 protein (New England BioLabs) and the resulting antiserum was used.

### Immunoblot analysis

*B. pertussis* clinical strains were inoculated in modified SS medium with a starting optical density of 0.2 at 600 nm, and further cultured with shaking at 36°C. Total protein was extracted with SDS-lysis buffer, and culture supernatant (CS) proteins were precipitated with 10% TCA. Protein samples were subjected to SDS-PAGE, transferred to nitrocellulose membranes (Bio-Rad) and incubated with anti-BteA, anti-BtcA, anti-BopD [32], anti-ACT, anti-FHA, or anti-PT antiserum. Antigen-antibody complexes were visualized using horseradish peroxidase (HRP)-conjugated secondary antibody (Bio-Rad, Hercules, CA) and ECL Western Blotting Detection Reagents (GE Healthcare).

### DNA sequencing

The region between the *btcA* and *bteA* gene corresponding to positions 165122 to 168021 of *B. pertussis* Tohama (GenBank accession: BX640412) was amplified in vaccine-type and nonvaccine-type clinical strains with the appropriate primers and sequenced. Sequencing reactions were carried out with the BigDye Terminator v3.1 Cycle Sequencing Kit (Applied Biosystems, Foster City, CA), and the products were sequenced on an ABI PRISM 3130*xl* Genetic Analyzer (Applied Biosystems).

### Transcriptional analyses

Total RNA was isolated using the RNeasy Mini Kit (QIAGEN, Hilden, Germany) and treated with RNase-free DNase (Promega) to degrade contaminating DNA. Reverse transcriptase-PCR (RT-PCR) was performed with bteA RT-R and btcA RT-R primers (Table S1) using the TAKARA One Step RNA PCR Kit (AMV, TAKARA Bio Inc.). PCR was performed with the following conditions: one cycle of 50°C for 30 min, 95°C for 2 min; 25 cycles of 95°C for 30 s, 58°C for 30 s, 72°C for 1 min; and a final incubation at 72°C for 10 min. Primer sets, bteA RT-F/bteA RT-R and btcA RT-F/btcA RT-R, were used for *bteA* and *btcA* amplification, respectively (Table S1). Products were analyzed by electrophoresis on a 1.5% agarose gel. Reverse transcriptase was omitted from the negative control reaction mixtures.

For quantitative RT-PCR (qRT-PCR), 5 µg of RNA was reverse transcribed into cDNA using the SuperScript First-Strand Synthesis System (Invitrogen, Carlsbad, CA) with random hexamer primers. Relative levels of total *bteA* and *recA* transcripts were determined using TaqMan probes (bteA- and recA-probes, Table S1) and *Premix Ex Taq*TM (Perfect Real Time, TAKARA Bio Inc.) with the ABI PRISM 7500 Sequence Detection System (Applied Biosystems). The qRT-PCR conditions were 30 s at 95°C, followed by 40 cycles of 95°C for 15 s and 60°C for 1 min. The expression of *recA* was used as an internal control [45]. All samples were run in triplicate and *bteA* transcript (P1 + P2 transcripts) was normalized to the *recA* transcript for each sample. The *bteA* IS*481a*-promoter transcript (P2 transcript) was determined using a TaqMan probe (IS481-bteA probe). The qRT-PCR conditions were 30 s at 95°C, followed by 40 cycles of 95°C for 15 s and 55°C for 1 min. The ratio of P2 transcript to total *bteA* transcript (P2 transcript/P1 + P2 transcripts) was estimated from four independent experiments. The regions amplified by qRT-PCR are shown in Figure 6A.

### Mapping transcriptional start sites

5′ rapid amplification of cDNA ends (5′-RACE) was performed using 5′-Full RACE Core Set (TAKARA Bio Inc.) according to the manufacturer's instructions. Reverse transcription was executed at 55°C using a 5′ phosphorylated RT primer (bteA-RT, Table S1). The first PCR used primers bteA-S1 (S1) and bteA-A1 (A1) primers, and bteA-S2 (S2) and bteA-A2 (A2) for the second (Table S1). PCR products were cloned into the pT7Blue T-vector (Novagen, Madison, Wis.) and transformed into *E. coli* XL1-Blue, which were plated on LB agar plates. Several clones were sequenced. The transcription start site of *btcA* was located using 5′-RACE with five primers, btcA-RT (5′ phosphorylated primer), btcA-S1 (S1), btcA-A1 (A1), btcA-S2 (S2) and btcA-A2 (A2) (Table S1).

### Generation of BteA mutants

Four BteA mutants, Δ*bteA*-BP155, Δ*bteA*-BP157, ΔIS*481*-BP155 and +IS1*481*-BP157, were constructed by homologous recombination as described previously with minor modifications [30] (Figure 6A).

*BteA-deficient mutants*: A 2.2-kbp DNA fragment containing a 5′ portion of the *bteA* gene was amplified by PCR with the B1-bteA and B2-bteA primers (Table S1) using the *B. pertussis* Tohama genomic DNA as the template. The PCR product was cloned into the pDONR221 vector (Invitrogen) to obtain pDONR-*bteA* by means of adaptor PCR and site-specific recombination techniques with the Gateway Cloning System (Invitrogen). Inverse PCR was then carried out with R1-bteA and R2-bteA primers (Table S1) using circular pDONR-*bteA* as the template. The R1-bteA and R2-bteA primers contained a BamHI site. The resulting PCR product was digested with BamHI and self-ligated to obtain pDONR-Δ*bteA*, which contained a 178-bp deletion around the 5′ region of *bteA*. pDONR-Δ*bteA* was mixed with pABB-CRS2 [46] to obtain pABB-Δ*bteA* using the Gateway Cloning System. pABB-Δ*bteA* was then introduced into *E. coli* SM10λ*pir* and transconjugated into streptomycin (SM)-resistant *B. pertussis* BP155 (vaccine-type) and BP157 (nonvaccine-type) clinical strains. The resultant mutant strains were designated Δ*bteA*-BP155 and Δ*bteA*-BP157.

*IS*481*-deletion mutant*: pABB-*bteA* was constructed from pDONR-*bteA*. pABB-*bteA* was introduced into *E. coli* SM10λ*pir* and transconjugated into SM-resistant *B. pertussis* vaccine-type BP155. The resultant mutant strain was designated ΔIS*481*-BP155.

*IS*481*-insertion mutant*: a 3.2-kbp DNA fragment (*bteA*+IS*481*) containing the *bteA* 5′-UTR and IS*481a* was amplified with the B1-bteA and B2-bteA primers (Table S1) using *B. pertussis* BP155 genomic DNA as the template. pABB-*bteA*+IS*481* was constructed from pDONR-*bteA*+IS*481* and transconjugated into SM-resistant *B. pertussis* nonvaccine-type BP157 via *E. coli* SM10λ*pir*. The resultant mutant strain was designated +IS*481*-BP157.

### Statistical analysis

The Student's *t*-test was employed. A value of *P*<0.05 was considered statistically significant.

### Nucleotide sequence accession number

The IS*481a* sequence was deposited in the DDBJ/EMBL/GenBank nucleotide sequence databases under accession number AB473880.

## Supporting Information

Figure S1 **High secretion of BteA protein in** ***Bordetella bronchiseptica*** **.** *B. bronchiseptica* (BB R05), *B. pertussis* BP155 (vaccine-type) and BP157 (nonvaccine-type) were cultured in modified SS medium for 24 h. Total protein extracted from the bacterial cells (Cell) and culture supernatants (CS) was separated by SDS-PAGE followed by silver staining (left panel). Immunoblots were incubated with anti-BteA, anti-BtcA or anti-BopD antiserum (right panel). For BteA detection, 0.5 µg of total protein (for Cell) and 5 µl of CS were loaded in the indicated lanes. The amount of total protein loaded was one-twentieth of that in Figure 2, and the loaded CS volume was one-fortieth of that in Figure 3.
(TIF)

Figure S2 ***In vitro*** **transcription-translation analysis of a** ***bteA*** **5′-UTR deletion series.** (A) *bteA* 5′-UTR deletion genes were PCR-amplified using *B. pertussis* BP155 (vaccine-type) as the template. Proteins were synthesized using the WakoPURE System (Wako Pure Chemical Industries, Ltd.). The 5′-UTR deletion genes harbored the T7 promoter at their 5′ end. (B) Expression of BteA protein in an *in vitro* transcription-translation system (WakoPURE System). The synthesized product was analyzed with immunoblots using anti-BteA antiserum. NC, negative control. (C) A predicted stem-loop structure in the 5′-UTR of *bteA* mRNA (P2 transcript). The RNA secondary structure was analyzed by CentroidFold (http://www.ncrna.org/centroidfold). The schematic shows a simplified map. TIR, translation initiation region.
(TIF)

Table S1 **Primers and probes in this study.**
(XLS)
