# Supplementary figures and images for: Differential Expression of Type III Effector BteA Protein Due to IS481 Insertion in Bordetella pertussis
Source: PLoS One. 2011 Mar 10;6(3):e17797. doi: 10.1371/journal.pone.0017797 (PMC3053399; doi:10.1371/journal.pone.0017797)

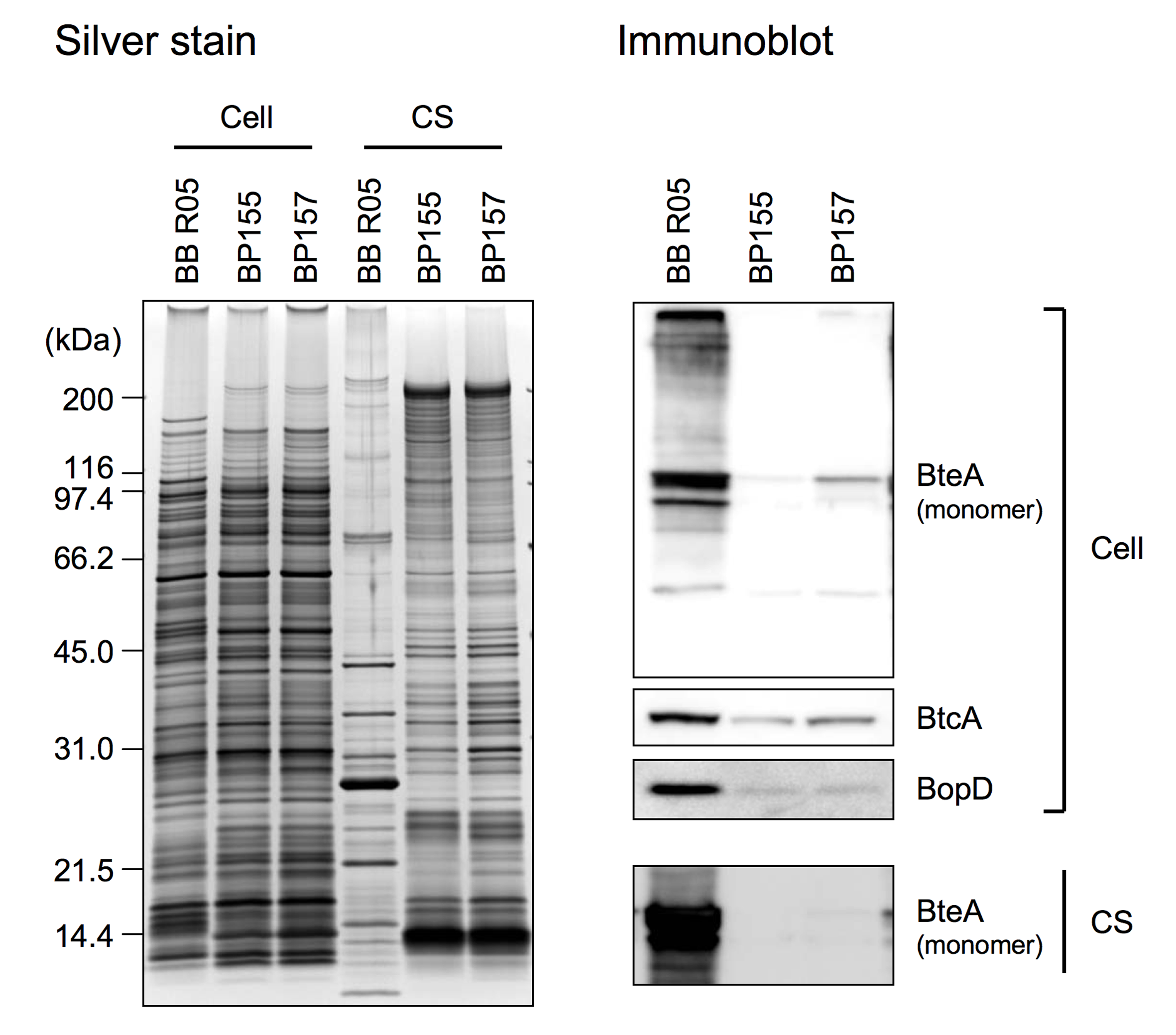

Supplement: Figure S1 — High secretion of BteA protein in Bordetella bronchiseptica . B. bronchiseptica (BB R05), B. pertussis BP155 (vaccine-type) and BP157 (nonvaccine-type) were cultured in modified SS medium for 24 h. Total protein extracted from the bacterial cells (Cell) and culture supernatants (CS) was separated by SDS-PAGE followed by silver staining (left panel). Immunoblots were incubated with anti-BteA, anti-BtcA or anti-BopD antiserum (right panel). For BteA detection, 0.5 µg of total protein (for Cell) and 5 µl of CS were loaded in the indicated lanes. The amount of total protein loaded was one-twentieth of that in Figure 2, and the loaded CS volume was one-fortieth of that in Figure 3. (TIF) [file pone.0017797.s001.tif]

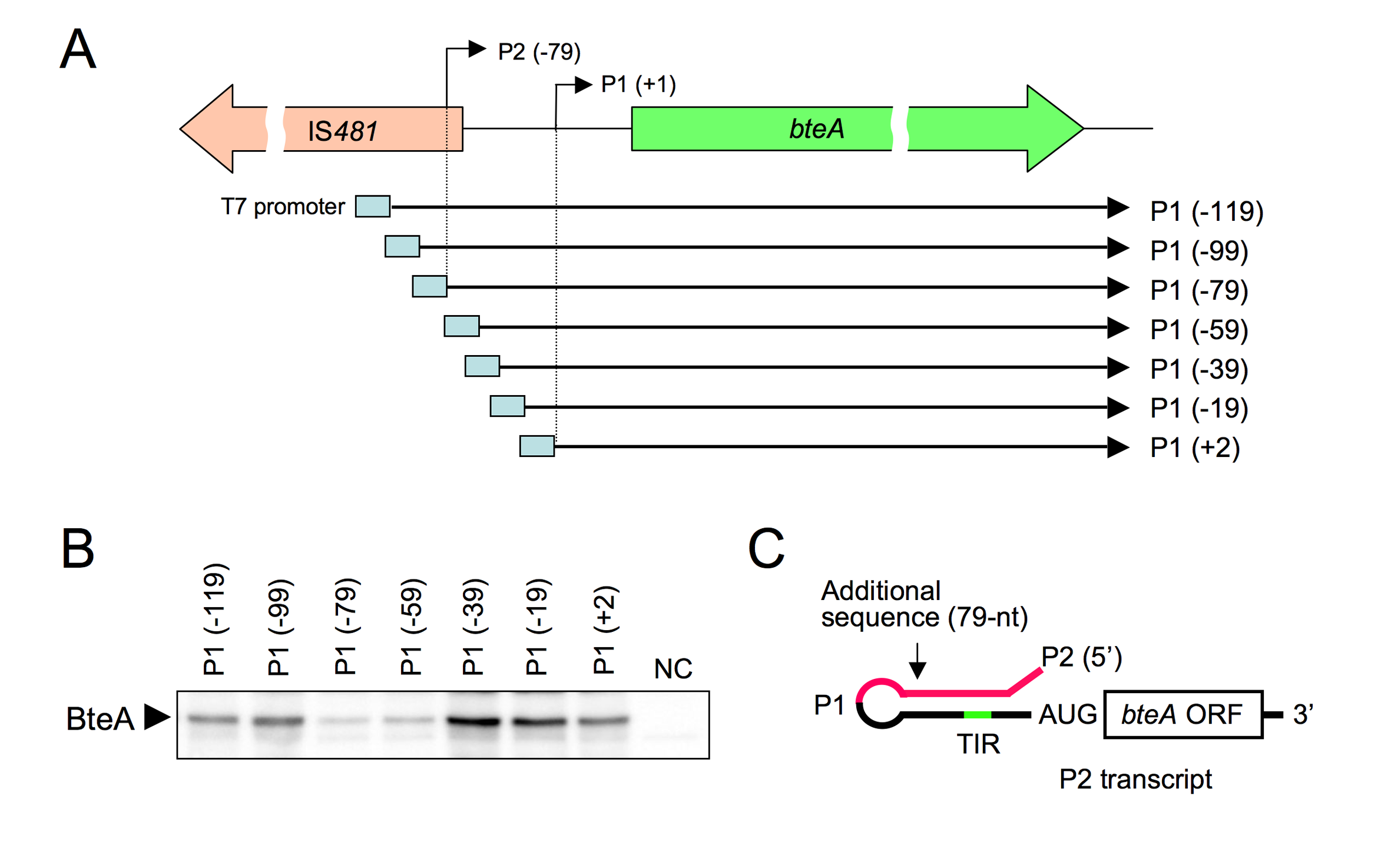

Supplement: Figure S2 — In vitro transcription-translation analysis of a bteA 5′-UTR deletion series. (A) bteA 5′-UTR deletion genes were PCR-amplified using B. pertussis BP155 (vaccine-type) as the template. Proteins were synthesized using the WakoPURE System (Wako Pure Chemical Industries, Ltd.). The 5′-UTR deletion genes harbored the T7 promoter at their 5′ end. (B) Expression of BteA protein in an in vitro transcription-translation system (WakoPURE System). The synthesized product was analyzed with immunoblots using anti-BteA antiserum. NC, negative control. (C) A predicted stem-loop structure in the 5′-UTR of bteA mRNA (P2 transcript). The RNA secondary structure was analyzed by CentroidFold (http://www.ncrna.org/centroidfold). The schematic shows a simplified map. TIR, translation initiation region. (TIF) [file pone.0017797.s002.tif]
